# Analysis of Off-Design Performance and Thermal–Fluid–Structural Coupling Characteristics of an Adjustable Air Ejector

**DOI:** 10.3390/ma19020294

**Published:** 2026-01-11

**Authors:** Yingwen Zhang, Liru Yan, Jingxian Zhang, Suxia Ma, Wenlong Guo

**Affiliations:** 1 College of Electrical and Power Engineering, Taiyuan University of Technology, Taiyuan 030024, China18435138742@163.com (J.Z.); m15033010093@163.com (W.G.); 2 Taiyuan Boiler Group Co., Ltd., Taiyuan 030008, China; yanlirua@163.com

**Keywords:** adjustable air ejector, thermal–fluid–structural (TFS) coupling, needle opening, thermal deformation, thermal stress

## Abstract

Systematic investigation into the structural integrity of adjustable ejectors, particularly concerning thermal–fluid–structural (TFS) coupling, is currently lacking. Utilizing the Workbench platform, this study performs unidirectional steady-state TFS coupling numerical simulation of the adjustable air ejector under off-design conditions to systematically analyze its internal flow characteristics and structural mechanical responses across various needle openings. The results show that thermal load is the dominant factor governing the ejector’s structural stress and deformation. The overall deformation is primarily characterized by axial elongation, with the maximum thermal deformation localized at the ejector’s exit section. The nozzle exit is identified as the primary structural weak point, exhibiting the highest local stress, which peaks at 196.8 MPa when the needle opening is minimized. Shock train structures extending from the nozzle’s divergent section into the mixing chamber, coupled with the axial displacement of the needle, significantly influence the ejector’s thermal deformation and thermal stress. Based on the thermally dominated stress mechanism identified, this study proposes a composite nozzle design utilizing a nickel-plated Invar alloy substrate. This material fully leverages Invar alloy’s low thermal expansion to mitigate thermal stress and deformation while the nickel plating ensures corrosion resistance, thereby significantly enhancing the nozzle’s mechanical properties and operational reliability in thermal environments. The findings of this analysis are applicable to off-design evaluations under unidirectional steady-state coupling conditions, providing a valuable reference for the structural design and strength optimization of similar ejectors operating in high-temperature, unsteady environments.

## 1. Introduction

**Figure 1:**
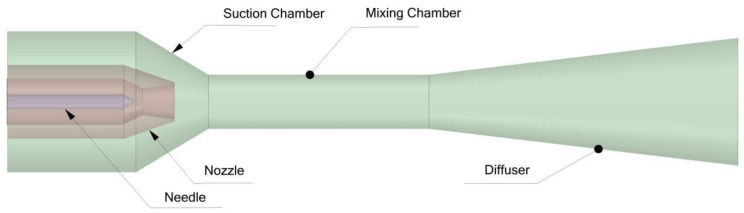
Schematic configuration of the adjustable ejector.

Ejectors, functioning as devices for fluid mixing and pressure boosting of low-pressure fluids, exhibit significant advantages in fields such as industrial waste heat utilization, refrigeration, and fuel cells due to their structural simplicity and absence of moving parts [1,2,3,4,5,6]. The fundamental operating principle relies on a high-pressure motive fluid entraining a low-pressure fluid. During the mixing process, the two fluids exchange momentum and energy, resulting in a mixed fluid at an intermediate pressure. To enhance operational performance under off-design conditions, adjustable ejectors equipped with a needle mechanism have been developed. Through axial displacement of the needle, the effective flow area of the Laval nozzle throat is modulated, thereby achieving precise control over the motive fluid flow rate [7,8,9,10,11,12,13]. Figure 1 illustrates the schematic configuration of the adjustable ejector. Its primary components include the nozzle, needle, suction chamber, mixing chamber, and diffuser.

Research on ejectors has primarily centered on establishing theoretical models and simulating performance. The theoretical modeling framework has evolved continuously, ranging from the one-dimensional model proposed by Keenan and Neumann [14,15] and Munday’s “choking” theory [16], to the two-dimensional “shock circle definition” model developed by Zhu et al. [17]. Furthermore, numerical simulations have been extensively utilized to elucidate the internal flow field structures [18,19]. However, most existing studies have focused on hydrodynamic performance [20,21,22]. Research remains insufficient regarding the multi-physics coupling challenges faced by ejectors under actual operating conditions, with a particular lack of systematic analysis concerning their structural integrity and TFS coupling characteristics. During operation, components must withstand fluid pressure loads, as well as forces from intense mixing or impact in localized regions. Consequently, ensuring sufficient structural strength is paramount for safe and reliable operation. Particularly under off-design conditions, shock train structures frequently emerge from the nozzle’s divergent section to the mixing section. This flow phenomenon, accompanied by intense energy dissipation, serves as the primary source of localized high-temperature gradients, which subsequently induce significant thermal stress through wall heat conduction. This process—wherein kinetic energy is converted into thermal energy, which in turn drives structural deformation—constitutes a quintessential TFS coupling mechanism. To date, research on the structural mechanical response of adjustable ejectors under complex loading remains insufficient, particularly regarding the characteristics of local stress concentrations induced by geometric complexity and multi-physics coupling effects. Therefore, verifying ejector strength cannot rely solely on the permissible stress of materials as the acceptance criterion. Instead, stress classification and verification must be conducted in accordance with the principles specified in ASME Boiler and Pressure Vessel Code, Section VIII, Division 2. This study investigates an adjustable air ejector by performing a unidirectional TFS coupling numerical simulation via the Workbench platform. It thoroughly explores internal flow characteristics, thermal deformation, and stress distribution patterns across different materials and operating conditions, providing a theoretical foundation for the safe design and performance optimization of ejectors.

## 2. Numerical Model

### 2.1. Geometric Model and Simplification

**Figure 2:**
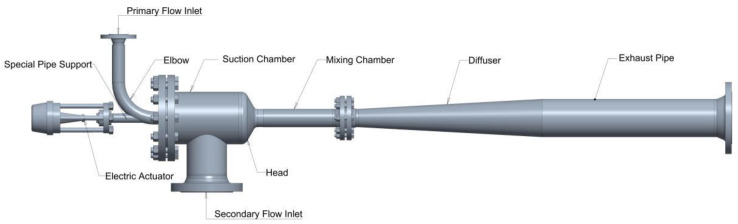
Geometric model of the adjustable air ejector.

The geometric model of the adjustable air ejector established in this study is illustrated in Figure 2. The structural dimensions of key components are shown in Table 1. To minimize the working fluid’s energy loss and mitigate the impact of the fluid jet on the needle, a bent pipe configuration is employed to change the air intake direction. An irregular pipe seat is integrated at the bent pipe opening to realize the integrated arrangement of local reinforcement and the sealing structure. The end of the needle is connected to an electric actuator, which controls its axial displacement for modulating the flow area of the nozzle throat. The nozzle section is connected via a flange bolt group to facilitate disassembly and replacement.

**Figure 3:**
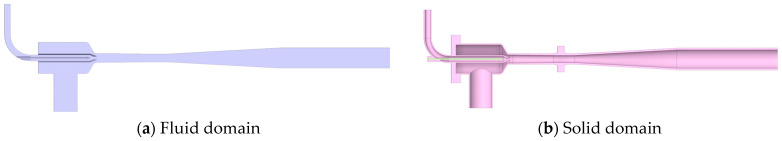
Simulation model of adjustable air ejector.

Considering the actual operating conditions and the need for simplification, the physical model underwent the following processing: the actuator, which has a negligible impact on the ejector body’s structural strength, is excluded; minor geometric features such as bolts, fillets, and chamfers are omitted. Given the symmetry of the ejector structure and constraint conditions, the Design Modeler 2021 R2 preprocessing software was utilized to generate a mirrored model. The fluid domain and solid domain were separately extracted through the volume extraction function, serving as the simulation models for the flow field and static analysis, respectively, as shown in Figure 3.

### 2.2. Computational Method

**Figure 4:**
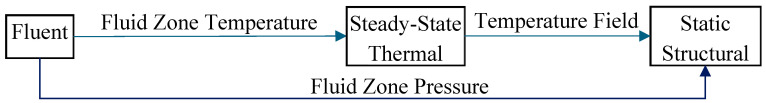
Implementation process of the coupled heat-fluid-solid analysis.

For the TFS coupling analysis of the adjustable air ejector, the flow field is calculated first. The resulting wall pressure and temperature fields are then applied as loads to the solid domain for structural analysis. Given that in the operation of the ejector, the impact of the flow field on solid deformation is small, and the feedback effect of solid deformation on the flow field distribution can be ignored. Moreover, as this study focuses on elucidating the global thermal stress distribution patterns driven by flow-induced heating rather than precisely simulating localized flow details under extreme deformation, a unidirectional coupling strategy is adopted, as it adequately fulfills the research objectives. The specific implementation process is shown in Figure 4.

### 2.3. Governing Equations

In the TFS coupling mechanical model, the continuity equation, momentum equation, and energy equation in the fluid domain are solved simultaneously with the static and dynamic equations in the solid domain. At the resulting coupled interface, the displacement (*r*), heat flux (*q*), temperature (*T*), stress (*τ*), and other parameters of the fluid and the solid shall satisfy the equality condition, which is expressed as the following coupled governing equations:

(1) $$\left\{\begin{array}{l} n\cdot \tau_{f}=n\cdot \tau_{s}\\ r_{f}=r_{s}\\ q_{f}=q_{s}\\ T_{f}=T_{s} \end{array}\right.$$

where *n* represents the interface normal vector, the subscript *f* denotes the fluid and the subscript *s* denotes the solid.

### 2.4. Mesh Generation and Boundary Condition Setup

#### 2.4.1. Fluid Domain

The fluid domain of the ejector was discretized, employing near-wall inflation layers to accurately resolve boundary layer gradients. To ensure grid independence, five mesh configurations with element counts of 1,025,314; 2,132,004; 3,361,723; 4,346,313; and 6,616,298 were generated and evaluated. The analysis indicates that the mass flow rates at the inlet and outlet converge when the element count reaches 3,361,723. Subsequent increases in mesh density yielded negligible deviations in the calculated results. Consequently, the mesh consisting of 3,361,723 elements was selected to balance computational efficiency with accuracy.

Based on the fluid properties, the SST k-omega turbulence model was selected. Pressure inlet boundary conditions were applied to the motive and entrained fluid inlets at 1.3 MPa and 0.13 MPa, respectively. The outlet was defined as a pressure outlet at 0.215 MPa. All walls were treated as smooth, no-slip, and adiabatic boundaries. Steady-state simulations were performed using a pressure-based implicit solver with pressure–velocity coupling. To verify the numerical model’s reliability, validation was performed by comparing the simulated entrainment ratio against the design value at the 100% opening condition. The simulation yielded an entrainment ratio of 0.98 against the design value of 1.0, corresponding to a relative error of 2%. This deviation falls well within the acceptable engineering tolerance (<5%), thereby confirming the model’s reliability.

#### 2.4.2. Solid Domain

To ensure the mesh independence of the structural stress and deformation results, local mesh refinement was performed on critical regions, such as the nozzle throat and needle support. Five sets of structural models with mesh counts of 188,258, 252,018, 389,564, 402,521, and 956,382 were generated for comparative calculation. By evaluating the peak stress and maximum deformation across these various mesh densities, the results demonstrate that the variations in these parameters remained below 3% upon further refinement. Consequently, a mesh count of 252,018 was selected for the solid domain to achieve an optimal balance between computational efficiency and numerical accuracy.

In the structural analysis, the ejector’s outer wall is assumed to be covered by a thermal insulation layer, and the convective heat transfer coefficient of the outer wall is set to 1 W/(m^2^·K). The physical loads acting on the structure include the fluid pressure and temperature fields mapped onto the inner wall from the fluid dynamic calculations. A standard gravity load is also applied to the model. Kinematic constraints were applied as follows: the axial displacement of the fluid inlet pipe section, the outlet pipe section, and the end face of the irregular pipe seat were constrained to zero (*δ_axial_* = 0). Additionally, the normal displacement of the symmetry plane was fixed at zero (*δ_normal_* = 0). The design pressure for the adjustable air ejector is 1.3 MPa, and the design temperature is 30 °C. The main body of the ejector is constructed from S30408 stainless steel, and the nozzle component is made of Invar alloy.

## 3. Results and Discussion

### 3.1. Flow Field Characteristics Analysis

#### 3.1.1. Variation in Performance Parameters with Opening

Table 2 presents the flow field boundary conditions under different operating conditions. Table 3 details the performance parameters of the ejector across various needle openings. The analysis indicates that as the opening decreases from 100% to 90%, the pressure increase ratio remains stable at the design value of 1.65, demonstrating robust pressure-boosting stability. However, further reduction in the opening triggers a performance transition: the pressure increase ratio declines, while the entrainment ratio increases. Specifically, at 70% opening, the pressure increase ratio drops to 1.14, whereas the entrainment ratio rises to 1.41. This phenomenon is attributed to the reduced effective throat area at lower openings, which significantly constrains the motive fluid mass flow rate. Since the magnitude of reduction in motive flow exceeds that of the entrained fluid, the result is a net increase in the entrainment ratio and a simultaneous decrease in the pressure increase ratio.

#### 3.1.2. Internal Flow Field Structure Under Design Conditions

**Figure 5:**
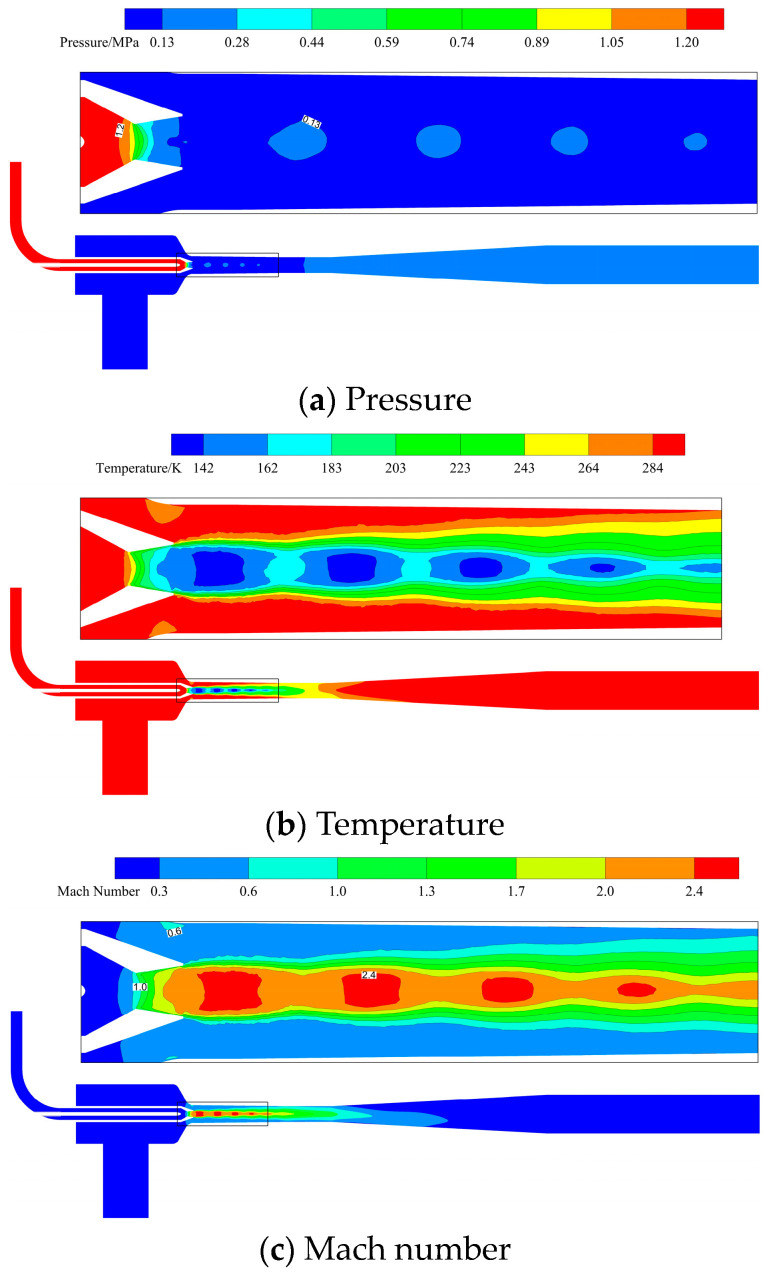
Contour plot of the flow field on the symmetry plane of the ejector.

Figure 5 shows the flow field contour on the ejector’s symmetry plane at the 100% opening (design condition). Under this condition, the motive fluid maintains a stable flow state prior to entering the nozzle, with an initial pressure of approximately 1.3 MPa and a temperature of about 302 K. In the convergent section, the flow velocity gradually increases due to the reduction in the cross-sectional area, accompanied by corresponding decreases in pressure and temperature. The flow reaches sonic speed (Ma = 1) at the throat. Upon entering the divergent chamber, the fluid undergoes further isentropic expansion and accelerates to supersonic speed (Ma > 1). A shock train structure appears at the nozzle exit, where both pressure and temperature drop further. Driven by the pressure difference and the entrainment effect, the entrained fluid is drawn into the mixing chamber [23,24,25,26,27,28,29]. Here, it undergoes intense momentum and energy exchange with the motive fluid. As the entrained fluid enters the mixing chamber, its pressure and temperature decrease while the velocity increases due to the contraction of the flow area. The high-speed motive fluid jet forms a distinct core flow region in the center of the mixing chamber, continuously accelerating and entraining the surrounding low-speed fluid. The two streams achieve complete mixing by the exit of the mixing chamber. Subsequently, the mixed fluid enters the diffuser, where kinetic energy is gradually converted into pressure energy. At the ejector’s outlet, the fluid parameters stabilize, reaching a final pressure of approximately 0.215 MPa and a temperature of about 302 K.

#### 3.1.3. Internal Flow Field Structure Under Different Openings

**Figure 6:**
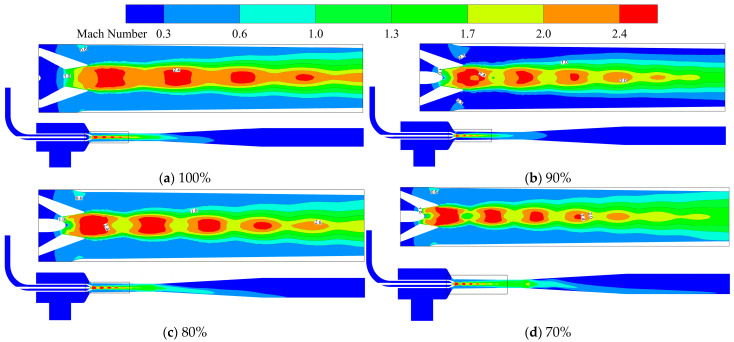
Contour plot of Mach number distribution on the symmetry plane of the ejector under different opening conditions.

**Figure 7:**
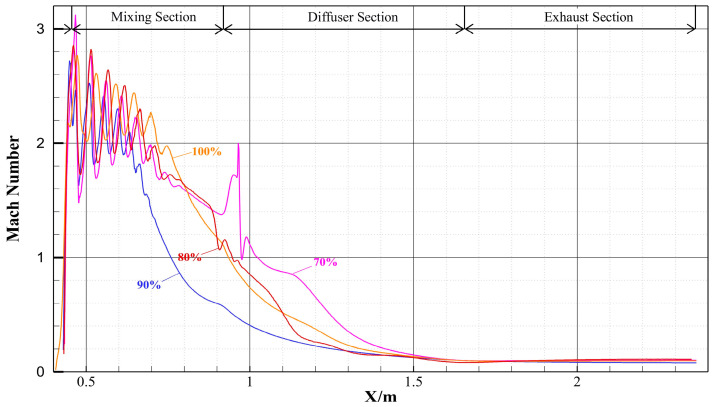
Variation curve of Mach number along the central axis of the ejector under different opening conditions.

Figure 6 shows the Mach number contours on the symmetry plane of the ejector under different openings. Figure 7 presents the variation curves of fluid Mach number along the central axis of the ejector under different openings. Under all openings, there are distinct shock wave structures inside the ejector, whose influence range extends from the divergent chamber of the nozzle to the mixing chamber, characterized by significant axial gradient in fluid Mach number. As the opening gradually decreases, the axial displacement of the needle causes a change in the throat flow area, leading to a modification of the internal flow field structure. Specifically, the position, intensity of the shock wave, and the range of the supersonic region all undergo changes. At 100% opening, the needle does not participate in flow regulation. The fluid velocity increases uniformly along the nozzle axis, with the average Mach number reaching 1 at the throat, and further accelerating to supersonic state in the divergent chamber. The shock wave structure continues to develop to approximately two-thirds of the mixing chamber When the opening decreases, the axial movement of the needle has a significant impact on the internal flow field structure of the nozzle. On one hand, an obvious high-pressure and low-velocity recirculation zone is formed at the needle tip due to flow separation; as the opening decreases and the throat area contracts, the scope of this vortex zone gradually expands. On the other hand, the position of the minimum flow area of the nozzle advances with the reduction in the needle opening, causing the fluid to accelerate to sonic speed earlier and enter the supersonic flow state faster in the divergent chamber. By further comparing the shock wave characteristics under different openings with Figure 7, it can be seen that under the condition of 80% and 70% openings, the shock wave length is significantly extended, the shock wave intensity is enhanced, and two distinct continuous shock train structures are formed in the flow channel.

### 3.2. Thermal Deformation and Stress Distribution

**Figure 8:**
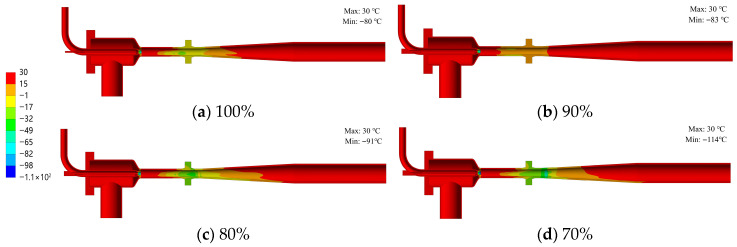
Temperature distribution of the ejector structure under different opening conditions (Unit: °C).

**Figure 9:**
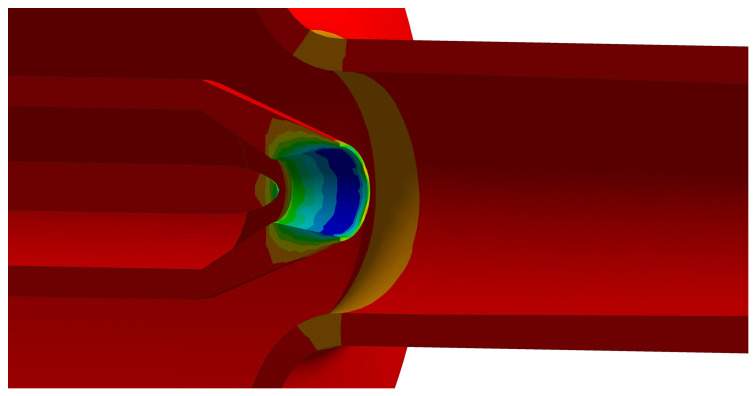
Temperature distribution at the nozzle needle tip of the ejector under 70% opening condition.

Figure 8 shows the structural temperature distribution of the ejector under various opening conditions, while Figure 9 details the specific temperature profile at the needle tip for the 70% opening case. As evidenced by the figures, the nozzle component consistently exhibits the minimum temperature across all openings. This region also displays the steepest temperature gradient, with a maximum temperature differential of 144 °C between the nozzle inlet and outlet. Secondary low-temperature zones are observed in the conical head and the mixing chamber. Correlating these thermal patterns with the flow field structure in Figure 6 reveals that the conical head contains a local vortex, while the mixing chamber houses a complex shock train structure. These flow phenomena, coupled with the complex local geometry, collectively govern the observed temperature distribution. Furthermore, as the needle opening varies, a significant temperature gradient forms in the needle tip region (as shown in Figure 9). The spatial extent of this gradient region expands as the opening decreases, indicating that the needle component is subjected to intense thermal loads during the regulation process.

**Figure 10:**
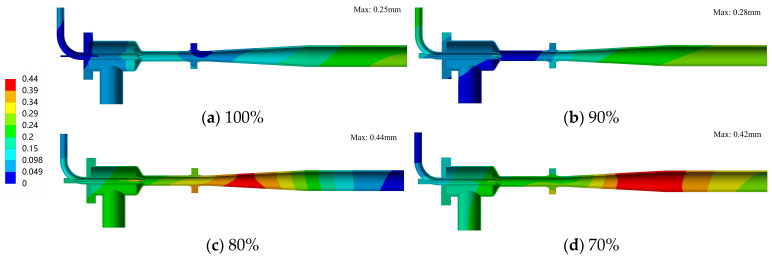
Deformation distribution of the ejector structure under different opening conditions (Unit: mm).

Figure 10 shows the magnitude of structural deformation across different openings, while Table 4 details the variations in the relative distance between the needle and the throat. As indicated in Figure 10, the maximum deformation observed across all conditions is 0.44 mm. Driven by thermal expansion, the maximum deformation is localized at the diffuser exit, with the overall structural response primarily characterized by axial elongation. Furthermore, the deformation magnitude intensifies as the needle opening decreases. Notably, at 80% and 70% openings, the generation of a secondary shock wave within the diffuser induces a local maximum deformation in that specific region. Additionally, since the needle serves as the core regulating component, its relative distance from the nozzle throat directly governs flow characteristics. Table 4 reveals that at the 100% opening (where the needle is inactive), the needle-to-throat distance decreases by 0.037 mm. Conversely, at other opening settings, this distance increases by a maximum of 0.06 mm. This dimensional variation remains within the system’s permissible tolerance and exerts a negligible impact on the ejector’s flow regulation performance.

**Figure 11:**
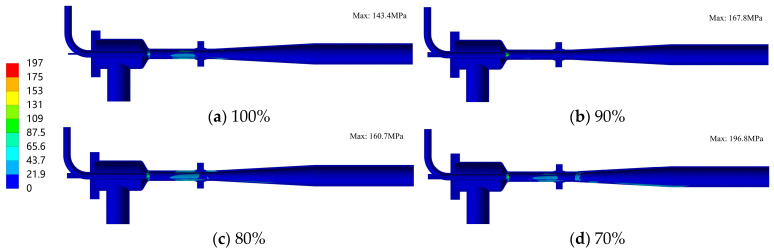
Stress distribution of the ejector structure under different opening conditions (Unit: MPa).

**Figure 12:**
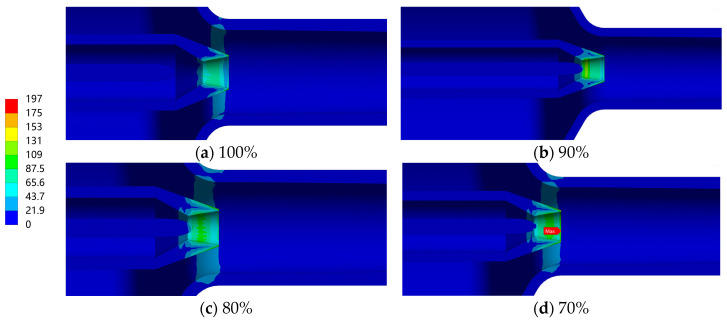
Stress distribution in the nozzle region under different opening conditions (Unit: MPa).

Figure 11 shows the stress distribution of the ejector structure under different openings, and Figure 12 presents the stress distribution in the nozzle region. As can be seen from the figures, under all openings, the maximum stress value of the ejector is 196.8 MPa, which all occurs at the nozzle outlet region. Moreover, as the opening decreases, the maximum stress value gradually increases. In addition to the nozzle, stress concentration also exists in regions such as the conical head, needle tip, and mixing chamber. However, their stress levels are relatively low, all not exceeding 42 MPa. The stress distribution described above indicates that the nozzle component is the primary structural weak point in the ejector. In contrast, the stress levels of other parts are relatively low, and their structural safety meets the service requirements.

### 3.3. Measures for Reducing the Maximum Stress of the Nozzle Component

As shown in Figure 11, the maximum structural stress is consistently concentrated within the nozzle component across various opening conditions. This region is characterized by significant temperature gradients and compact geometry. Furthermore, during the needle adjustment process, micro-deformations in the nozzle can compromise the device’s regulation accuracy. To address these issues of stress concentration and thermal deformation, Invar alloy—renewed for its extremely low coefficient of thermal expansion—is proposed as the nozzle substrate material. This selection aims to enhance the nozzle’s mechanical properties and operational reliability. To further augment corrosion resistance within the compressed air environment, precision nickel plating is applied to the surface. This composite design effectively minimizes flow channel deformation while ensuring structural stability and durability in thermal environments. Additionally, the temperature dependence of material properties is incorporated to enhance the accuracy of the thermal–mechanical coupling simulation. Table 5 lists the mechanical properties of candidate materials at the design temperature, while Table 6 compares the maximum structural stress values for different nozzle materials at the 70% opening condition. The results demonstrate that the utilization of Invar alloy effectively mitigates structural stress concentration.

### 3.4. Stress Distribution of the Ejector Structure Under Different Loads

**Figure 13:**
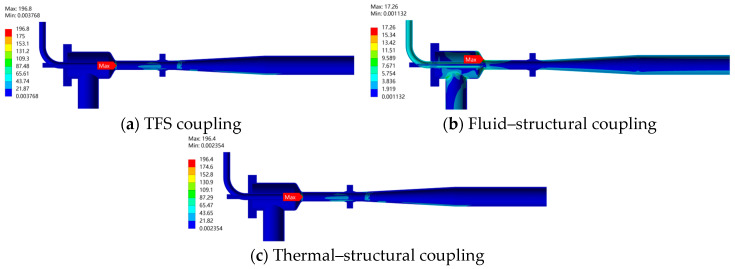
Multi-load Stress Distribution of the Ejector Structure at 70% Opening (Unit: MPa). (**a**) TFS coupling; (**b**) Fluid–structural coupling; (**c**) Thermal–structural coupling.

Figure 13 shows the multi-load stress distribution of the ejector structure under 70% opening. As can be seen from the stress contour shown in Figure 13b, under the action of fluid–structural coupling, the maximum stress of the ejector occurs in the conical head region with a value of 17.26 MPa. The stress distribution considering only thermal loads is basically consistent with that under the TFS coupling effect. The maximum stress values and their locations of occurrence are relatively close, indicating that thermal loads play a dominant role in determining the structural stress state, while the fluid–structural coupling effect has a relatively limited impact on the overall stress distribution. Comprehensive analysis indicates that thermal load is the main factor affecting the structural strength of the ejector. Therefore, in structural design and strength evaluation, thermal load analysis should be given priority to ensure the safe and reliable operation of the equipment under complex thermal–mechanical conditions.

### 3.5. Strength Check

To accurately evaluate the strength reliability of the ejector structure under TFS coupling loads, stress classification and check are performed on the nozzle outlet region with the most significant stress concentration at various openings in accordance with ASME BPVC Section VIII. The total stress is decomposed into primary membrane stress, primary membrane + bending stress, and primary + secondary stress. A stress linearization path is defined along the wall thickness direction at the location of maximum stress. Taking 70% opening as an example, the linearization check results are shown in Table 7. In the check, the material of the nozzle component is Invar alloy. At −100 °C, the yield strength of this material is 445 MPa, and the tensile strength is 729 MPa. The analysis shows that each stress component meets the allowable limits specified by the standard.

## 4. Conclusions

Through TFS coupling numerical simulation, the flow characteristics and structural field characteristics of the adjustable ejector under off-design conditions have been systematically studied, and the main conclusions are as follows:

(1) Within the region from the nozzle divergent chamber to the mixing chamber of the ejector, there exist complex supersonic flows and shock train structures in the internal flow. The axial displacement of the needle causes the sonic position to move forward and the range of the supersonic region to change, while significantly affecting the intensity and position of the shock wave.
(2) Thermal load is the decisive factor affecting the structural stress and deformation of the ejector. Internal shock train structures, extending from the nozzle’s divergent section to the mixing chamber, induce localized high-temperature gradients through intense energy dissipation. These gradients, coupled with structural irregularities, drive significant local stress concentrations. The observed peaks in wall temperature and thermal stress align closely with the thermal load localization predicted by the energy dissipation mechanism. The maximum thermal deformation occurs at the end of the ejector, and the nozzle outlet region is the main load-bearing weak link with relatively high local stress. The maximum stress value increases as the opening decreases, reaching 196.8 MPa. Although stress concentration exists in parts such as the conical head and the needle tip, the stress levels are all below 42 MPa.
(3) A nozzle material of the nickel-plated Invar alloy substrate is proposed. By fully leveraging the low thermal expansion characteristic of Invar alloy to reduce thermal stress and thermal deformation, and ensuring corrosion resistance through the nickel-plated surface, the mechanical properties and operational reliability of the nozzle are effectively improved.

These conclusions provide a direct reference for the design optimization of high-reliability ejectors. To further enhance engineering fidelity, subsequent research will involve experimental validation of the numerical results to provide more robust practical guidance and mitigate the risk of structural failure under intense thermal loads.

## Figures and Tables

**Table 1:** Structural dimensions of key components of the ejector.

Structural Parameters	Value
Nozzle inlet diameter/mm	39.10
Nozzle throat diameter/mm	15.50
Nozzle outlet diameter/mm	22.20
Mixing chamber diameter/mm	52.64
Mixing chamber length/mm	404.30
Diffuser length/mm	1511.00
Diffuser outlet diameter/mm	131.50

**Table 2:** Flow field boundary conditions under different operating conditions.

	Motive Inlet	Entrained Inlet	Outlet
100% opening	1.3 MPa	0.13 MPa	0.215 MPa
90% opening	1.3 MPa	0.13 MPa	0.215 MPa
80% opening	1.3 MPa	0.13 MPa	0.165 MPa
70% opening	1.3 MPa	0.13 MPa	0.148 MPa

**Table 3:** Performance parameters of the ejector under different openings conditions.

	100% Opening	90% Opening	80% Opening	70% Opening
motive inlet flow rate (t/h)	2.00	1.76	1.60	1.43
entrained inlet flow rate (t/h)	1.98	1.22	2.03	2.01
outlet flow rate (t/h)	3.98	2.98	3.63	3.44
entrainment ratio	1.00	0.70	1.27	1.41
pressure increase ratio	1.65	1.65	1.27	1.14

**Table 4:** Variation in the distance between the nozzle needle and throat under different opening conditions.

Opening	100%	90%	80%	70%
The variation in the distance between the needle and the throat/mm	−0.037	+0.042	+0.06	+0.054

**Table 5:** Main parameters of different materials at design temperature.

Material	Density/Kg dm^−3^	Coefficient of Thermal Expansion/K^−1^	Elastic Modulus/GPa	Thermal Conductivity/W (m K)^−1^	Yield Strength/MPa	Tensile Strength/MPa
304	7.93	1.6 × 10^−5^	199	15	205	520
304H	7.9	1.63 × 10^−5^	199	17	205	520
304L	7.9	1.6 × 10^−5^	199	15	180	480
Invar alloy	8.1	1.2 × 10^−5^	140	10.2	276	448

**Table 6:** Maximum structural stress values under different nozzle materials at 70% opening condition.

Material	304	304L	304H	Invar Alloy
Maximum stress value/MPa	242.1	299	290	196.8

**Table 7:** Stress linearization check results.

Evaluation Type and Stress Limits	Calculated Stress/MPa	Allowable Stress/MPa	Conclusion
Primary membrane stress $P_{m}\leq S_{m}$	163.5	223	Qualified
Primary membrane stress + Primary bending stress $P_{l}+P_{b}\leq 1.5S_{m}$	196.6	334.5	Qualified
Primary stress + Secondary stress $P_{l}+P_{b}+Q\leq 3S_{m}$	196.8	669	Qualified

## Data Availability

The original contributions presented in this study are included in the article. Further inquiries can be directed to the corresponding author.
